# Majocchi's Granuloma after Topical Corticosteroids Therapy

**DOI:** 10.1155/2014/507176

**Published:** 2014-10-23

**Authors:** Fu-qiu Li, Sha Lv, Jian-xin Xia

**Affiliations:** The Second Hospital of Jilin University, Changchun, Jilin 130000, China

## Abstract

Majocchi's granuloma (MG) is an unusual but not rare dermatophyte infection of dermal and subcutaneous tissues. Dermatophytes usually result in the infections of hair, epidermis, and nail, and are rarely involved in deep cutaneous and subcutaneous tissues. Now it is considered that MG includes two forms: one is a small perifollicular papular form and the other is a deep subcutaneous nodular form; the front one mainly occurs in healthy individuals and the latter one usually presents in immunocompromised hosts. The clinical manifestations of MG are many and varied, except the common presentations of erythema, papule and nodules, and Kaposi sarcoma-like and molluscum-like lesions have been reported in literatures (Kim et al. (2011), Bord et al. (2007), and Lillis et al. (2010)). This characteristic induces the difficulty of diagnosis, and thus it is so important and necessary to make direct microscopical and histological examinations. We describe a case of MG over the face in a patient who had been treated with topical corticosteroids over a long time.

## 1. Case Report

**Figure 1:**
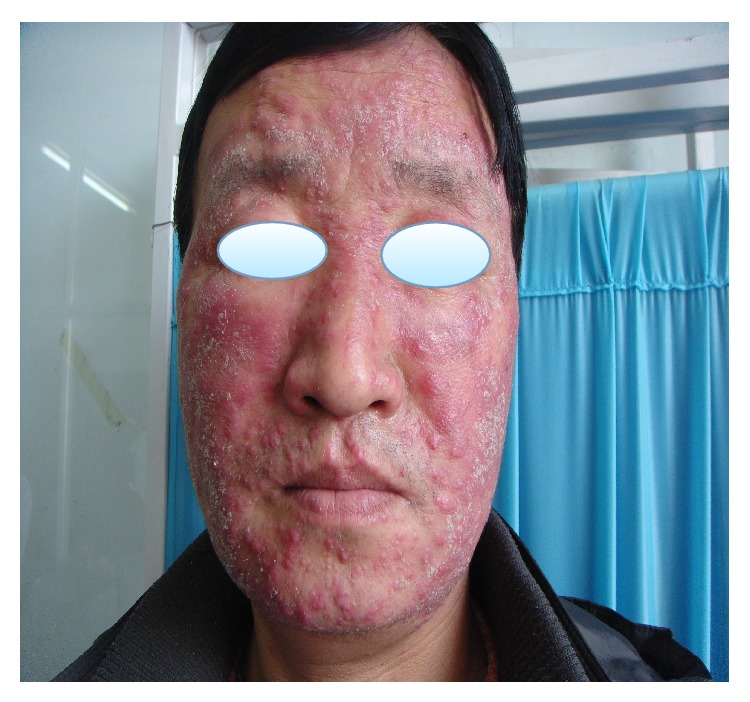
Erythema, papule and nodules, and desquamation over the whole face.

**Figure 2:**
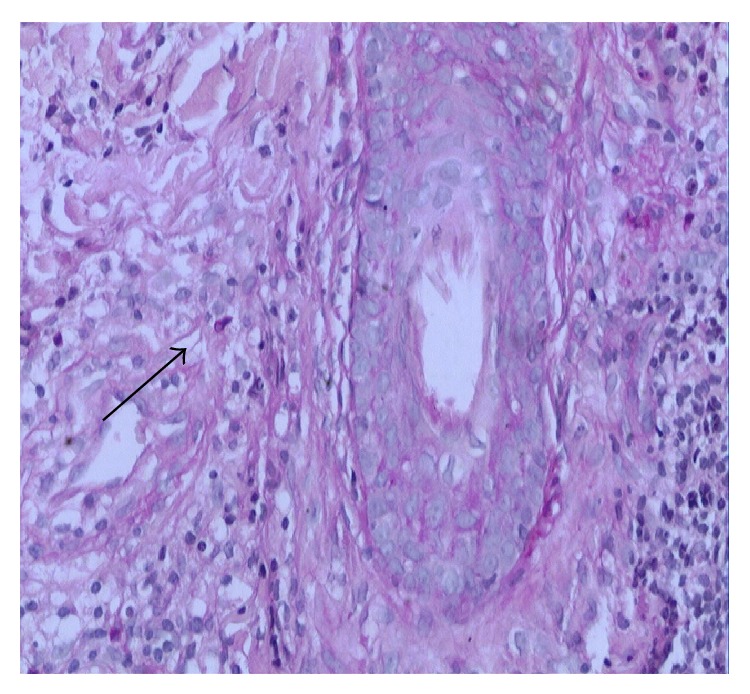
One spore was noted in the dermis which was stained with D-periodic acid-Schiff (D-PAS).

**Figure 3:**
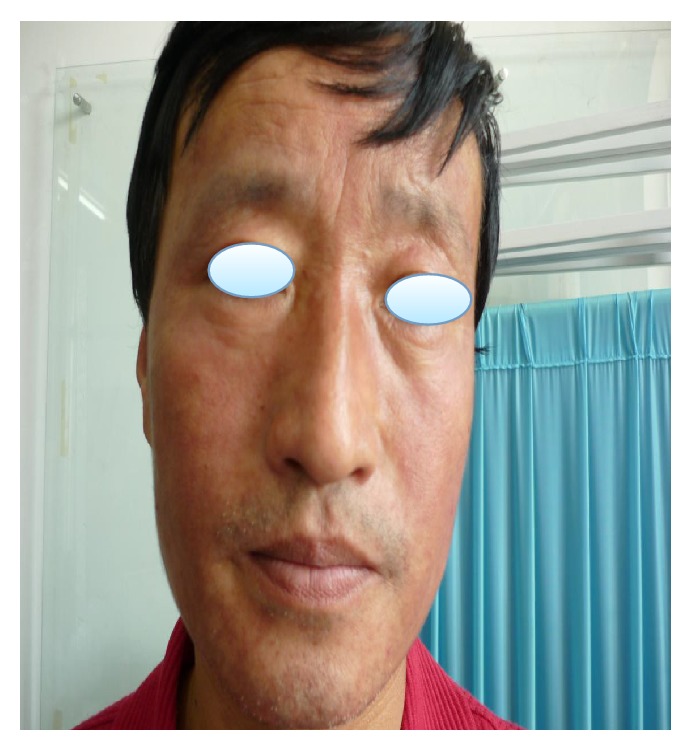
The lesions completely disappeared.

A 46-year-old male patient presented with one-year history of erythema, papule and nodules, and desquamation over his whole face, accompanied with pruritus. He had consulted doctors in some private clinics, was diagnosed with eczema and solar dermatitis and was treated with topical corticosteroids and systemic antibiotic or antiallergic drugs for several months intermittently. The patient had been treated with topical corticosteroids including fluocinolone, dexamethasone ointment, and mometasone furoate off and on for about three months. The lesions had been improved at one time and then grown worse. Skin examination reveals multiple variably sized red and firm nodules on his face accompanied with swelling; some were associated with surrounding erythema and desquamation on an erythematous base (Figure 1). The patient denied any history of trauma like shaving of the face or previous cutaneous fungal infections such as tinea pedis, onychomycosis, or fungal infections of other regions. There were no other systemic diseases. The HIV test was negative. KOH examination of the discharge materials reveals distinct hyphae. Histological examination of a punch biopsy specimen taken from the left face showed cell granulomas with lymphocytes, neutrophils, and monocytes around the follicle and vessels in the dermis, and yet no hyphae and spores were detected. Only one spore was noted in the dermis which was stained with D-periodic acid-Schiff (D-PAS) (Figure 2). The fungal culture of biopsy specimen yielded the growth of spreading white colonies with a cottony surface and red reverse pigment. Based on clinical, KOH examination, and histopathological and culture findings, MG caused by* Trichophyton rubrum* was identified. Systemic treatment was with itraconazole 200 mg twice daily and topical sertaconazole olamine for 4 weeks; after that, the dose of itraconazole reduced to 100 mg twice daily for the following 4 weeks; then the lesions completely disappeared (Figure 3).

## 2. Discussion

MG was first described as a kind of deep granulomatous trichophytosis by Professor Majocchi in 1883 [1]. The initiating factors are usually divided into two types in literatures; one is thought to be physical trauma which is induced by shaving, pricking, and other external forced like that and the other is due to immunosuppression caused by immunosuppressive therapy or autoimmune diseases that had been reported in literatures [2–8]. These two factors lead to disruption of the follicle in the dermis and subcutaneous tissue when hosts are infected by dermatophytes which usually invade epidermis. The most common causative pathogen is* Trichophyton rubrum*, and* Microsporum canis* and* Aspergillus fumigatus* have also been reported in literatures [7, 9]. The pathogens generally exist in stratum corneum because keratinous material can potentially provide a substrate for the organism [10]. It is believed that keratin is carried by the severe inflammation into the dermis [3] and then provides a suitable living environment for the organism.

In our report, the patient had no history of trauma such as shaving of the face or previous cutaneous fungal infections and had no immunosuppression; in that way what is the initiating factor? Immunosuppressive drugs include corticosteroids, vincristine, cyclophosphamide, azathioprine, and tacrolimus, alone or in combination. Jacobs [11] thought that misapplication of topical corticosteroids over a long period can produce Majocchi granuloma. It was reported that MG was made by applying betamethasone ointment for 4 months by Meehan [12]. When it occurs to the patient, it might be eczema or allergic diseases at first, and he consulted doctors in private clinics and was given topical corticosteroids over a long time; then the host was occasionally infected by dermatophytes; the application of corticosteroids initially suppressed the inflammatory component associated with dermatophytes while simultaneously accelerating dermatophyte growth [9].

Owing to the variety of MG's manifestations, it is necessary to make examinations including direct microscopical, histological examinations and the fungal culture. When the patient came to our clinic for diagnosis and treatment, these diagnoses including corticosteroid-dependent dermatitis, lupus miliaris disseminates, acne rosacea, fungal infective dermatoses, and skin tumour were considered by us. After a series of examinations, MG was diagnosed as being caused by* Trichophyton rubrum* which was identified by the features of fungal culture.

Reviewing the past literatures about MG's treatment, we can find that systemic antifungals such as itraconazole or terbinafine are a must because of the deep location of the infection. The duration of therapy does not have the same standard, from 3 weeks to 1 year in the literatures, and it takes more time for immunosuppressed individuals in general. For the treatment of Majocchi's granuloma, topical antifungals are usually ineffective [13]. Direct microscopical examinations of the discharge materials reveal obviously hyphae in our case that indicates the organism lives not only in deep cutaneous and subcutaneous tissues but also in epidermis, so it was assumed that topical antifungals are effective for this patient.
